# Hepatic GCGR is required for the superior weight loss and metabolic effects of a structurally related analogue of the dual GCGR/GLP‐1R agonist survodutide in mice

**DOI:** 10.1111/dom.70359

**Published:** 2025-12-12

**Authors:** Fen Long, Tenagne D. Challa, Vissarion Efthymiou, Manuel Klug, Thomas Klein, Heike Neubauer, Christian Wolfrum, Carla Horvath

**Affiliations:** ^1^ Institute of Food, Nutrition and Health ETH Zürich Schwerzenbach Switzerland; ^2^ Department of Cardiometabolic Diseases Research Boehringer Ingelheim Pharma GmbH & Co KG, Biberach Germany; ^3^ Nanyang Technological University (NTU) Singapore

## 1 BACKGROUND

Novel peptide‐based anti‐obesity and anti‐diabetic drugs have revolutionized obesity and metabolic disease treatment.^1^ The addition of the glucagon receptor (GCGR) component to glucagon‐like peptide 1 receptor (GLP‐1R) agonism has improved weight loss efficacy compared to GLP‐1R stimulation alone and may provide additional cardiometabolic benefits.^2^, ^3^ While the effects on appetite regulation and glucose control through GLP‐1R agonism are well established, the specific role of GCGR agonism in promoting weight loss and metabolic health is less defined.

GCG regulates multiple metabolic pathways affecting lipid, glucose and amino acid homeostasis,^4^ primarily through hepatic GCGR signalling. However, the use of GCG as a monotherapy is limited by its hyperglycaemic effects. As a dual GCGR/GLP‐1R agonist, survodutide has demonstrated robust weight loss effects in individuals with obesity, with preclinical and early clinical data suggesting potentially superior efficacy to semaglutide (GLP‐1RA) monotherapy.^2^, ^5^ Moreover, survodutide has shown significant improvements in metabolic dysfunction‐ associated steatohepatitis (MASH) and fibrosis.^5^, ^6^ These outcomes suggest that GCGR agonism provides direct metabolic advantages such as increased energy expenditure (EE), lipolysis, and hepatic fat clearance. However, the precise contribution of GCGR activation and the underlying target tissues remain unclear. Survodutide's potent effects on body weight reduction are attributable to the additive actions of simultaneous GCGR and GLP‐1R activation. While GLP‐1R activation suppresses appetite, only GCGR agonism increases EE in mice and humans,^7^ underscoring the potential of GCG on resting metabolic rate.^2^ Using the dual GCGR/GLP‐1R agonist BI‐456908 and the selective GLP‐1R agonist semaglutide, we show that the dual agonist achieved superior weight loss efficacy by engaging hepatic GCGR without adversely affecting glucose control. We further demonstrate that hepatic GCGR signalling facilitates plasma and liver lipid clearance in response to the dual agonist. These findings highlight the crucial metabolic contributions of hepatic GCGR to the efficacy of combined GCGR/GLP1‐R activation.

## 2 METHODS

High‐fat diet‐induced obese wildtype (WT) or liver‐specific GCGR knockout mice were treated with BI‐456908 or semaglutide (s.c., daily for 28 days). BI‐456908 is a back‐up compound of survodutide, inducing comparable physiological effects but is not intended to advance for clinical trials as described previously^8^ and outlined in the Supporting Information Methods. Intraperitoneal glucose tolerance tests were performed after 21 days. Plasma parameters were measured at day 28. Liver triglycerides were extracted from liver samples and measured with a commercial kit. Detailed methods and reagents are found in the Supporting Information Methods.

## 3 RESULTS

**FIGURE 1:**
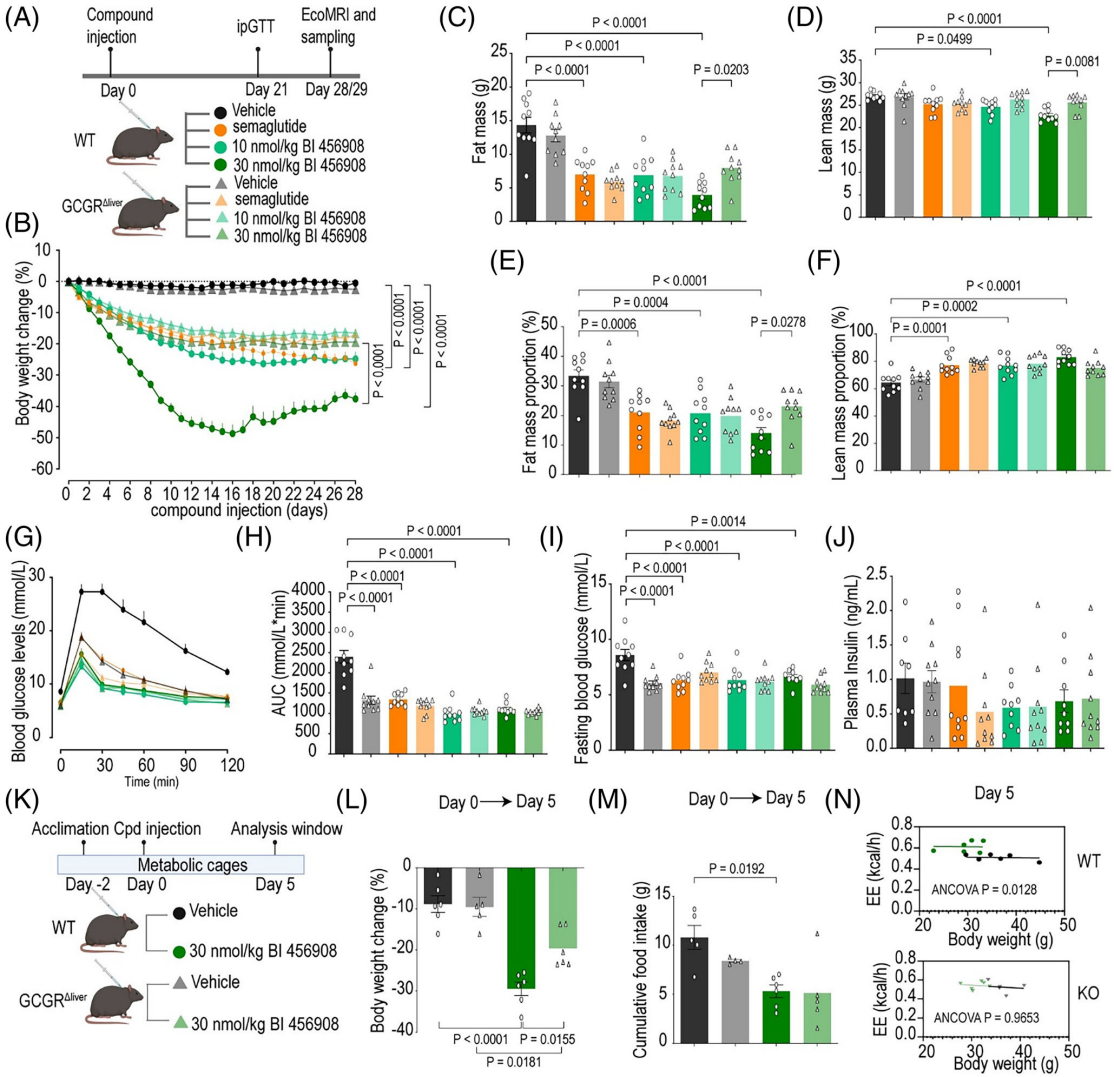
Metabolic improvements following 29 days of semaglutide or BI‐456908 treatment in DIO WT and hepatic GCGR KO mice. (A) Experimental design. (B) Percentage body weight change, daily s.c. injections (*n* = 10/group). (C–F) Body fat mass (C), lean mass (D), fat mass proportions (E), lean mass proportions (F) at D29 (*n* = 10/group). (G–I) Intraperitoneal glucose tolerance test (G) and (H) area under the curve at D21. (I) Fasting blood glucose levels at baseline (*n* = 9 for 30 nnmol/kg BI‐456908/WT group, *n* = 10 for other groups). (J) Plasma insulin at D29 (*n* = 8–10/group). (K) Experimental design for metabolic cages. (L) Percentage body weight change, daily s.c. injection (*n* = 5 for vehicle/LKO, *n* = 6 for other groups). (M) Cumulative food intake (*n* = 4–6/group after exclusion of three outliers identified by ROUT (*Q* = 1%) due to high fat diet crumbling in the metabolic cages). (N) Analysis of covariance (ANCOVA) of energy expenditure on D5 with body weight as a covariate (*n* = 5 for vehicle/LKO, *n* = 6 for other groups). Statistical significance indicates comparison between treated‐WT mice and WT controls or between treated‐WT and treated‐GCGR LKO mice. Statistical analysis was performed using two‐way ANOVA with Tukey's post hoc test. *p < 0.05. DIO, diet‐induced obese; GCGR, glucagon receptor; LKO, liver‐specific GCGR knockout; WT, wild type.

We employed the dual GCGR/GLP‐1R agonist BI‐456908, a structural and pharmacological analogue to survodutide^8^ as well as the GLP‐1R selective agonist semaglutide, to dissect the role of the glucagon component in weight loss and metabolic improvements. We utilized a liver‐specific GCGR knockout (LKO) mouse model to differentiate the effects mediated specifically by hepatic GCGR signalling (Figure 1A). Semaglutide (20 nmol/kg) resulted in ~20% weight loss in both diet‐induced obese (DIO) WT and LKO mice after 4 weeks of treatment (Figure 1B). BI‐456908 caused dose‐dependent weight loss in DIO WT mice. At 10 nmol/kg, WT mice lost ~20% weight, with a small difference between WT and LKO mice. At 30 nmol/kg, WT mice showed a substantial weight loss of ~40%. This pronounced weight loss efficacy of the high dose was significantly diminished in LKO mice, approximating the effects of semaglutide. These findings underscore the critical role of hepatic GCGR activation in the weight loss efficacy of BI‐456908 and its additive effects to GLP‐1R agonism. The weight reduction induced by semaglutide and BI‐456908 was primarily attributed to fat mass loss (Figure 1C), although some lean mass loss was observed in BI‐456908‐treated WT mice at the high dose (Figure 1D). Both agonists altered body composition, decreasing fat mass and increasing lean mass proportions (Figure 1E,F). However, at the high dose, BI‐456908 led to a more pronounced decrease in fat mass proportion in WT versus LKO mice, suggesting a depletion of fat stores to fuel energetic demands in the liver. Increased EE is commonly accompanied by a compensatory food intake, which maybe counteracted by the appetite suppressing effect of GLP‐1R agonism in the case of BI‐456908. The enhanced energetic demand is likely fuelled by adipose tissue lipolysis, apparent in the greater fat mass loss in BI‐456908 compared to semaglutide‐treated mice.

BI‐456908 ameliorated glucose tolerance and decreased fasting blood glucose levels at both doses comparably to semaglutide (Figure 1G–I). These improvements were comparable to those in LKO mice, demonstrating that BI‐456908 improves weight loss without compromising glucose homeostasis due to the effectiveness of the GLP‐1R component in improving glycaemic control. Fasting insulin levels were highly variable and not reduced in any group (Figure 1J). The weight loss induced by BI‐456908 reflects both reduced food intake and elevated EE (Figure 1K–N). Notably, the EE increase requires hepatic GCGR signalling, as both is evident in WT but blunted in LKO mice, consistent with prior reports that GCGR activation augments EE.^9^

**FIGURE 2:**
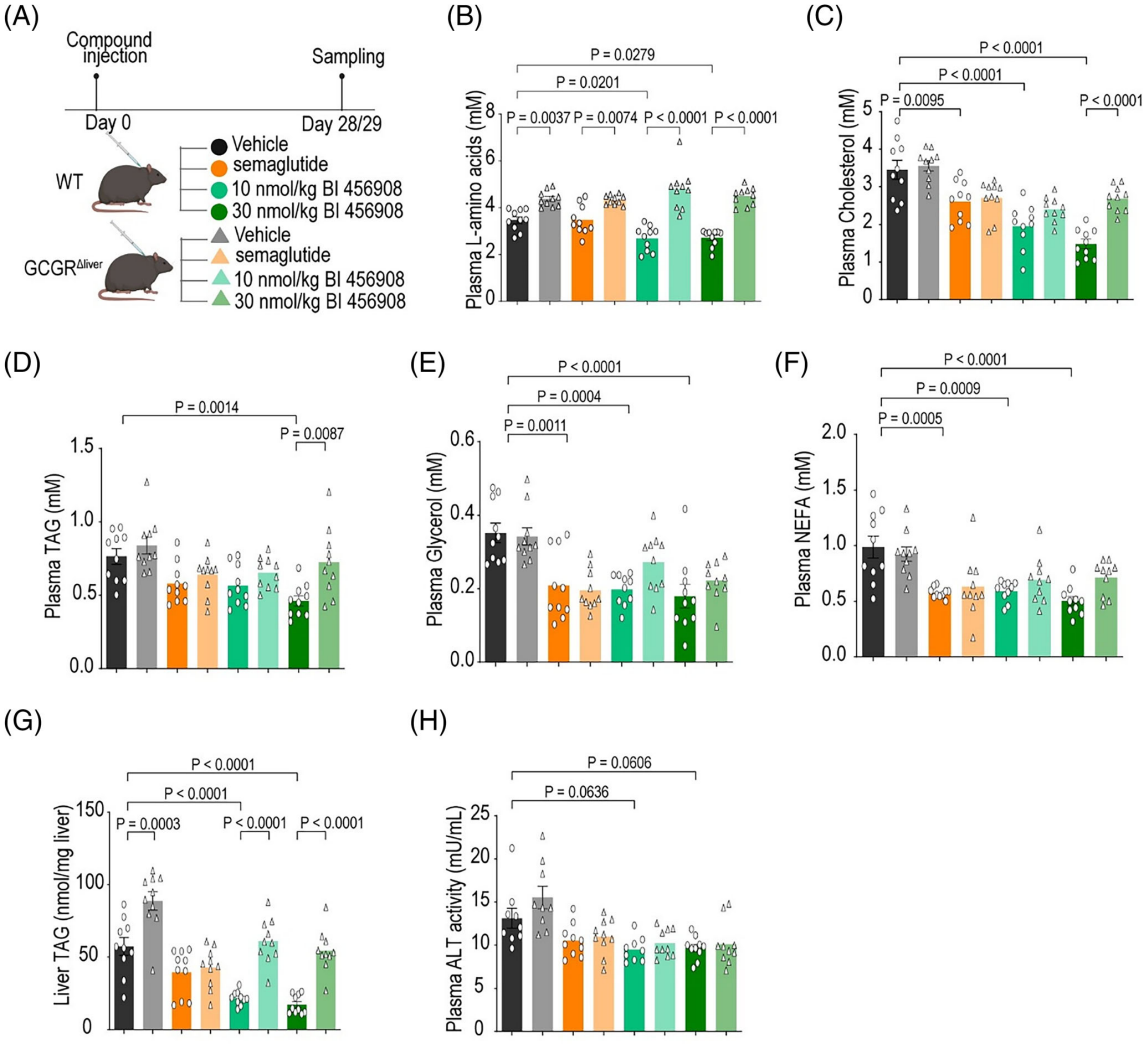
Metabolic improvements following 29 days of semaglutide or BI‐456908 treatment in DIO WT and hepatic GCGR KO mice. (A) Experimental design. (B–F) Plasma amino acids (B), cholesterol (C), TAG (D), glycerol (E), and non‐esterified fatty acids (NEFA) (F) levels at D29 (each *n* = 10/group). (G) Liver TAG levels at d29 (*n* = 10/group). (H) Plasma ALT at D29 (*n* = 9 for vehicle/WT; vehicle/LKO; 10 nmol/kg BI‐456908/WT group, *n* = 10 for other groups). Statistical significance indicates comparison between treated‐WT mice and WT controls or between treated‐WT and treated‐GCGR LKO mice. Statistical analysis was performed using two‐way Analysis of variance (ANOVA) with Tukey's post hoc test. *p < 0.05. DIO, diet‐induced obese; GCGR, glucagon receptor; LKO, liver‐specific GCGR knockout; TAG, triacylglycerol; WT, wild type.

BI‐456908 reduced circulating amino acid levels in WT but not in LKO mice, indicating hepatic GCGR‐dependent amino acid catabolism (Figure 2B). Semaglutide and BI‐456908 lowered plasma cholesterol, demonstrating GLP‐1R's benefits in plasma lipid clearance (Figure 2C). Hepatic GCGR activation further reduced cholesterol levels, evidenced by the differential effects of high‐dose BI‐456908 between WT and LKO mice. Moreover, only high‐dose BI‐456908 reduced plasma triacylglycerol (TAG) levels, which was abrogated in LKO mice (Figure 2D), indicating the metabolic advantages of hepatic GCGR activation. Both agonists similarly reduced plasma glycerol and free fatty acid levels (Figure 2E,F). Therefore, all treatment groups ameliorated dyslipidaemia, substantiating the systemic metabolic benefits and therapeutic potential of dual GCGR/GLP‐1R agonists. These lipid‐lowering effects may also stem from improved metabolic control linked to weight loss, higher metabolic activity in the liver or direct GCG‐controlled regulation of molecular processes. Accordingly, the dose‐dependent effects of BI‐456908 on plasma cholesterol levels might result from enhanced low‐density lipoprotein (LDL) cholesterol clearance as hepatic GCGR agonism promotes the degradation of proprotein convertase subtilisin/kexin type 9 (PCSK9), which controls LDL‐receptor internalization.^10^ GCGR^11^ stimulation enhances lipolysis and fatty acid oxidation in the liver, prompting us to evaluate its potential in reducing liver fat deposition, a known effect of survodutide treatment.^6^, ^8^ While semaglutide did not reduce liver TAG load, we could recapitulate enhanced TAG clearance in WT mice at both doses of BI‐456908, which were ineffective in LKO mice (Figure 2G). In contrast, vehicle‐treated LKO mice displayed higher liver TAG content compared to WT mice. Moreover, BI‐456908 effectively reduced plasma alanine transaminase (ALT) levels (Figure 2H), substantiating improved liver metabolic health, but this effect was independent of hepatic GCGR.

## 4 DISCUSSION

Concurrent activation of GCGR and GLP‐1R in different target tissues culminates in robust weight loss due to food intake‐dependent and ‐independent mechanisms. We show that hepatic GCGR signalling is essential for BI‐456908's additive weight loss effect and our data identify the liver as an integral component of systemic EE, which influences energy homeostasis to a physiologically relevant extent. These findings are consistent with those reported in global GCGR KO mice. Pocai et al. showed reduced body weight in response to dual GLP‐1R/GCGR agonism in WT mice but showed attenuated weight‐loss efficacy in global GCGR KO mice, indicating activation of both GLP‐1R and GCGR is required for the full anti‐obesity effect.^12^ In agreement, our prior work showed that a long‐acting GCGR agonist fails to increase EE and consequently fails to reduce body weight in hepatic GCGR KO^9^, highlighting the central role of hepatic GCGR in mediating weight loss mechanisms. In contrast, adipose tissue GCGR KO retained full EE and weight‐loss responses, arguing against adipose GCGR as the primary driver. Consistent with this model, BI‐456908 increased EE, lowered circulating amino acids, and reduced lipids in WT but not in hepatic GCGR KO mice, indicating hepatic GCGR‐dependent metabolic benefits.

The precise contributors of liver‐mediated EE are not fully elucidated, but hypoaminoacidemia and intact hepatic Farnesoid X receptor signalling were identified as prerequisites for the GCG‐induced increase in EE.^7^, ^13^ This corroborates the lipolytic activity of GCGR activation in the liver‐mediated downstream by phospholipase C and protein kinase A, stimulating lipolysis and mitochondrial fatty acid oxidation.^14^ Importantly, a physiological increase in circulating glucagon levels can boost hepatic mitochondrial oxidation in metabolic dysfunction‐ associated fatty liver disease (MAFLD) patients.^15^

Increased EE is intrinsically linked to higher mitochondrial respiration and substrate oxidation to generate a proton gradient across the inner mitochondrial membrane, which either powers ATP production or dissipates as heat through mitochondrial uncoupling via uncoupling protein 1 (UCP1).^16^ Because hepatocytes lack *Ucp1* expression, the increased EE following chronic GCGR activation likely drives ATP synthesis, raising the question of which specific metabolic processes operate at such high energy costs. Futile cycles are opposing chemical reactions that run simultaneously without generating a net product but waste ATP and may contribute to the observed hypermetabolic state. Futile lipid cycles constitute the continuous hydrolysis of TAGs into glycerol and free fatty acids (FFA) followed by the re‐esterification into TAGs. It is conceivable that glycerol and FFAs derived from white adipose tissue (WAT) lipolysis are re‐esterified in the liver and form a systemic futile lipid cycle, while the necessary ATP is delivered from oxidized FFA from the circulation and intrahepatic stores.^16^ The consequent removal of FFA and glycerol from the periphery could explain why high lipolytic rates in WAT of BI‐456908‐treated mice are not mirrored in elevated levels of these metabolites.

Results from clinical trials underline the additive efficacy of dual GLP‐1/glucose‐dependent insulinotropic peptide receptor (GIPR) and triple GLP‐1/GIPR/GCGR regarding weight loss and metabolic improvements. Notably, these effects are induced by different modes of action and increasing the possible combinations of targeting receptors will be beneficial to optimize therapeutic strategies for low responders or non‐responders on certain treatments. Retatrutide (GLP‐1/GIPR/GCGR) induces greater liver fat loss than tirzepatide (GLP‐1/GIPR), which is associated with a dose‐dependent increase in circulating levels of the ketone body β‐hydroxybutyrate.^17^ This biomarker for hepatic fatty acid oxidation provides evidence that liver GCGR engagement in humans promotes fat oxidation and ameliorates MASH beyond weight loss‐driven effects. Preclinical murine studies with dual GCGR/GLP‐1R agonist suggest that the effects on EE are partly mediated by FGF21 signalling.^18^ In humans, retatrutide does not increase plasma FGF21 levels, indicating potential species differences in the molecular mechanism underlying MASH improvements.^17^

In line with our study, data from survodutide treatment in humans show that GCGR activation does not adversely affect glycaemia and improves HbA1c levels.^3^ However, balancing the relative potencies of the GLP‐1R and GCGR components appears crucial to achieve beneficial outcomes on glucose control, as other GCGR/GLP‐1R dual agonists with more potent GCGR activity showed limited efficacy in improving glucose metabolism.^19^ Although muscle volume loss in response to retatrutide lies in the expected range for anti‐obesity medication,^20^ it remains unclear how potential hypoaminoacidemia affects muscles with dual GCGR/GLP‐1R agonism.

In conclusion, we demonstrate that hepatic GCGR activation is responsible and necessary for the substantial additive weight‐loss and metabolic effects of dual GCGR/GLP‐1R agonists, emphasizing the clinical relevance of GCGR agonism in the liver. It remains elusive to what extent this effect depends on EE or partly the anorectic effects of GCGR agonism. Yet, the metabolic processes underlying the increased energy demand remain unclear and warrant further investigation. Mechanistically, current evidence proposes that GCGR signalling in the liver drives mitochondrial fatty acid oxidation for ATP production, potentially fuelled by adipose tissue lipolysis and liver triglycerides, thereby increasing systemic EE.

## AUTHOR CONTRIBUTIONS

F.L., C.D.T., H.N., T.K., C.W. and C.H. designed the studies. C.W. and C.H. supervised the study. F.L., C.D.T., M.K. and C.H. performed experiments, data analysis and prepared figures. F.L., C.W. and C.H. wrote the manuscript. All authors edited and approved the manuscript.

## CONFLICT OF INTEREST STATEMENT

The authors meet criteria for authorship as recommended by the ICMJE. The authors did not receive payment related to the development of this publication. Boehringer Ingelheim was given the opportunity to review the manuscript for medical and scientific accuracy as well as intellectual property considerations. The study was supported and funded by Boehringer Ingelheim. BI‐456908 is a back‐up compound to survodutide.

## Supporting information


**Data S1.** Supporting Information

## Data Availability

All raw data for the displayed graphs are submitted in the Supporting Information.
