# Supplementary material for: Hepatic GCGR is required for the superior weight loss and metabolic effects of a structurally related analogue of the dual GCGR/GLP‐1R agonist survodutide in mice
Source: Diabetes Obes Metab. 2025 Dec 12;28(3):2437–42. doi: 10.1111/dom.70359 (PMC12890723; doi:10.1111/dom.70359)
Supplement: Supplementary file 1 — Data S1. Supporting Information [file DOM-28-2437-s001.pdf]

Supplementary Methods for
Hepatic GCGR is required for the weight loss and metabolic effects of a structural analogue of the dual GCGR/GLP1R agonist survodutide in mice

Fen Long[1], Tenagne D. Challa[1], Vissarion Efthymiou[1], Manuel Klug[1], Thomas Klein[2], Heike Neubauer[2], Christian Wolfrum[1,3], Carla Horvath[1*]

[1]Institute of Food, Nutrition and Health, ETH Zürich, Schwerzenbach, Switzerland
[2]Department of Cardiometabolic Diseases Research, Boehringer Ingelheim Pharma GmbH & Co KG, Biberach, Germany
[3]Nanyang Technological University (NTU), 50 Nanyang Avenue, Singapore

*Corresponding author: carla.horvath@ethz.ch

Extended Methods

Animal experiments and liver-specific GCGR KO mouse model

All animal procedures in this study were approved by the Cantonal Ethics Committee of the Veterinary Office of the Canton of Zurich. Except for metabolic cage experiments (see below), 2-5 littermates were housed together in ventilated cages at standard housing conditions (22 °C, 12 h reversed light/dark cycle, dark phase starting at 7am, 40% humidity), with ad libitum access to standard chow diet (18% protein, 4.5% fiber, 4.5% fat and 6.3% ashes) and water. GCGR floxed mice were generated by the Center for Transgenic Models, University of Basel, Switzerland. Hepatocyte-specific GCGR knockout mice were obtained by crossing GCGR floxed mice with Albumin-Cre (#035593, JAX) mice, resulting in Alb-Cre x GCGR fl/fl mice. No effect of the genotype was observed on viability. 6-8 weeks old liver-specific GCGR KO mice and Cre-negative littermates were challenged with high fat diet HFD, (60% kcal from fat, diet no. 3436, Provimi Kliba SA) for 16 weeks prior to compound treatment. No signs of toxicity were observed in response to semaglutide or BI 456908 treatment. One mouse in the BI was lost due to very rapid, profound weight loss that was likely affected by treatment-unrelated or additive causes. At the end of the study, animals were euthanized with a CO2 overdose.

Peptides Administration

BI 456908 and semaglutide were obtained from Boehringer Ingelheim. The compounds were dissolved in a phosphate buffer (50 mM, pH 7.0) with 5% mannitol, which also served as vehicle control. All peptides were administered at a volume of 5 mL/kg. The first dose was given in the morning (dark cycle for animals) of Day 0.

Details on compounds used: BI 456908 is a structural analogue to BI 456906 (survodutide), which is currently in phase III clinical trial for the treatment of obesity and MASH. BI 456908 was one of the candidate compounds for dual GCGR/GLP-1R agonism tested by Boehringer Ingelheim as published earlier[1], where BI 456906 (survodutide) was selected as candidate compound for clinical testing. In these studies, BI 456908 but not survodutide elicited elevated heart rates in mice at the highest dose of 100 nmol/kg BW in mice. We applied BI 456908 for mechanistic studies in genetically modified mice due to their comparable potencies to survodutide. The functional potency of BI 456908 is 0.92 nM for human GCGR and 0.61 for human GLP-1R nM as determined in Chinese ovary (CHO)-K1 cells expressing human GCGR or human GLP-1R using cAMP assays with compounds dissolved in assay buffer (140 mM NaCl, 3.6 mM KCl, 0.5 mM $NaH_2PO_4*2H_2O$, 0.5 mM $MgSO_4*7H_2O$, 1.5 mM $CaCl_2*2H_2O$, 10 mM HEPES, 5 mM $NaHCO_3$, 0.1% bovine serum albumin, 0.5 mM IBMX; pH 7.4)[1]. The potency for BI 456906 in the same system is 0.6 nM for GLP-1R and 0.52 nM for GCGR. Notably, the potencies of survodutide in 100% human plasma on HEK293 cells expression Cre-Luciferase under the human GCGR or GLP-1R receptor are 8.3 nM and 1 nM, respectively[2].

Body composition analysis and indirect calorimetry

Body composition measurements were obtained on conscious mice using quantitative nuclear magnetic resonance imaging (EchoMRI). Fat mass and lean mass were analysed using the Echo MRI1 14 software.

Indirect calorimetry and food intake were assessed using the Promethion Metabolic System (Sable Systems International). Mice were single-housed in Promethion metabolic cages with

ad libitum access to HFD and water. Baseline measurements were recorded for 2 days, followed by daily injections performed between 10-11 am. Food intake was measured continuously by the integrated gravimetric food hoppers of the Promethion system and expressed as cumulative intake. The EE ANCOVA analysis done for this work was provided by the NIDDK Mouse Metabolic Phenotyping Centers (MMPC, www.mmpc.org) using their Energy Expenditure Analysis page (http://www.mmpc.org/shared/regression.aspx) and supported by grants DK076169 and DK115255.

Intraperitoneal glucose tolerance tests

Intraperitoneal glucose tolerance test (ipGTT, 2g/kg BW) was performed after 21 days of treatment after four hours of fasting. Blood glucose concentrations were measured from the tail vein at timepoints 0, 15, 30, 45, 60, 90 and 120 minutes using a commercially available glucometer (ACCU-CHEK Aviva, Roche) with glucose strips. The area under the curve was calculated from the blood glucose concentrations measured between 0 and 120 minutes.

Plasma parameters

At the end of the study (day 29), cardiac blood was collected by cardiac after 4 hrs of fasting. Plasma was obtained by centrifugation at 8,000g for 20 min at 4°C. The following kits were used for plasma analyses insulin (ultra-sensitive mouse insulin ELISA kit, Crystal Chem, Cat#90080), Glycerol (Free Glycerol Reagent, Sigma-Aldrich, Cat#F6428), NEFA (NEFA assay kit, Wako NEFA kit, Cat#633-52001), TAG (TAG assay kit, SPINREACT, Cat#41033), total cholesterol (LabAssay™ Cholesterol kit, FUJIFILM Cat#635-50981), ALT activity (ALT activity assay, Sigma-Aldrich, Cat#MAK052), Amino acids (L-Amino Acid Assay Kit, Sigma-Aldrich, Cat# MAK490).

Hepatic TAG level determination

Liver samples (50-100 mg) were weighed and homogenized in 1 ml of isopropanol per 50 mg tissue. The homogenates were incubated with rotation before centrifugation at 2,000g for 10 min at 4°C to collect the supernatant. TAG levels were determined using a triglyceride assay kit (SPINREACT, Cat#41033) and normalized to liver sample weight.

Quantification and statistical analysis

Littermates were randomly assigned to treatment groups. Sample size was determined based on previous studies examining the effects of BI 456908 and survodutide on weight loss and metabolic parameters in diet-induced obese mice using comparable doses[1,2]. The exact animal number per group are indicated in the corresponding figure legends. One point corresponds to one animal. Data are represented as mean±SEM, unless indicated differently. Data were statistically analysed by ANOVA using GraphPad Prism 10 software using two-way ANOVA with Tukey's post hoc multiple comparison tests . $^*p<0.05$ was considered significant.

References

1. Thomas, L., Martel, E., Rist, W., Uphues, I., Hamprecht, D., Neubauer, H., and Augustin, R. (2024). The dual GCGR/GLP-1R agonist survodutide: Biomarkers and pharmacological profiling for clinical candidate selection. Diabetes Obes Metab *26*, 2368–2378. https://doi.org/10.1111/dom.15551.

2. Zimmermann, T., Thomas, L., Baader-Pagler, T., Haebel, P., Simon, E., Reindl, W., Bajrami, B., Rist, W., Uphues, I., Drucker, D.J., et al. (2022). BI 456906: Discovery and preclinical pharmacology of a novel GCGR/GLP-1R dual agonist with robust anti-obesity efficacy. Mol Metab *66*, 101633. https://doi.org/10.1016/j.molmet.2022.101633.
